# Variable 3’polyadenylation of *Wheat yellow mosaic virus* and its novel effects on translation and replication

**DOI:** 10.1186/s12985-019-1130-z

**Published:** 2019-02-20

**Authors:** Guowei Geng, Chengming Yu, Xiangdong Li, Xuefeng Yuan

**Affiliations:** Department of Plant Pathology, College of Plant Protection, Shandong Agricultural University; Shandong Province Key Laboratory of Agricultural Microbiology, No 61, Daizong Street, Shandong Province Tai’an, 271018 People’s Republic of China

**Keywords:** *Wheat yellow mosaic virus*, Variable polyadenylation, In vitro translation, In vitro replication, NIb

## Abstract

**Background:**

Polyadenylation influences many aspects of mRNA as well as viral RNA. variable polyadenylation at the 3' end have been reported in RNA viruses. It is interesting to identify the characteristic and potential role of 3' polyadenylation of *Wheat yellow mosaic virus* (WYMV), which has been reported to contain two genomic RNAs with 3' poly(A) tails and caused severe disease on wheat in East Asia region.

**Methods:**

3' RACE was used to identify sequences of the 3′ end in WYMV RNAs from naturally infected wheat by WYMV. In vitro translation assay was performed to analyze effect of UTRs of WYMV with or without 3’polyadenylation on translation. In vitro replication mediated by WYMV NIb protein were performed to evaluate effect of variable polyadenylation on replication.

**Results:**

Variable polyadenylation in WYMV RNAs was identified via 3′ RACE. WYMV RNAs in naturally infected wheat in China simultaneously present with regions of long, short, or no adenylation at the 3' ends. The effects of variable polyadenylation on translation and replication of WYMV RNAs were evaluated. 5’UTR and 3’UTR of WYMV RNA1 or RNA2 synergistically enhanced the translation of the firefly luciferase (*Fluc*) gene in in vitro WGE system, whereas additional adenylates had an oppositive effect on this enhancement on translation mediated by UTRs of WYMV. Additional adenylates remarkably inhibited the synthesis of complementary strand from viral genome RNA during the in vitro replication mediated by WYMV NIb protein.

**Conclusions:**

3' end of WYMV RNAs present variable polyadenylation even no polyadenylation. 3' polyadenylation have opposite effect on translation mediated by UTRs of WYMV RNA1 or RNA2. 3' polyadenylation have negative effect on minus-strand synthesis of WYMV RNA in vitro. Variable polyadenylation of WYMV RNAs may provide sufficient selection on the template for translation and replication.

**Electronic supplementary material:**

The online version of this article (10.1186/s12985-019-1130-z) contains supplementary material, which is available to authorized users.

## Background

Polyadenylation at the mature 3′ end of eukaryotic mRNAs plays a fundamental role in mRNA export to cytoplasm, effective translation and mRNA stability [1–3]. The cycle of polyadenylation and poly(A)-stimulated RNA degradation regulate the quality of gene expression in prokaryotes and eukaryotes [4, 5].

Viruses are obligate parasites and rely on living cells to complete their life cycles. RNAs of many DNA viruses and RNA viruses also have a 3′ poly(A) tail that is synthesized in a post-transcripitional manner, similar to mRNAs, or derived from a poly(U) sequence on the template strand [6–11]. The poly(A) tail of viral RNA may have at least two major functions mimicking roles of the stable poly(A) tails in eukaryotic mRNA. One of these is the control of RNA decay, which is associated with viral RNA stability [12, 13]; the other is the regulation of translation of viral proteins, which may be mediated synergistically by poly(A)-binding protein and initiation factor eIF4G [14, 15]. However, poly(A) tails of RNA viruses have different characteristics from those of eukaryotic mRNAs, including relatively shorter length and variable length [16, 17]. In addition to serving as the template for translation, RNA of RNA viruses also serves as the template for the synthesis of complementary the strand during replication [18–20]. Poly(A) length is related to the synthesis of complementary strand as well as virus infectivity [17, 21, 22]. It is implied that polyadenylation of RNA viruses could have more multi-level functions than that of mRNAs.

*Wheat yellow mosaic virus* (WYMV) causes yellow mosaic and dwarf symptoms on wheat and yield losses of 20–40% [23, 24]. WYMV was first identified in Japan [25] and has been subsequently reported in East Asia [24, 26, 27]. WYMV belongs to the genus *Bymovirus* in the family *Potyviridae*, whose members are 3′ polyadenylated and contain a 5′ genome-linked protein VPg [26]. WYMV has two positive single-strand RNAs. RNA1, 7.6 kb in size, encodes a polyprotein of 2404 amino acids that is subsequently split into coat protein and several putative nonstructural proteins such as VPg and NIb protein, which has RNA-dependent RNA polymerase activity. RNA2, 3.6 kb in size, encodes a polyprotein of 904 amino acids containing a putative proteinase and a 73kD polypeptide [26]. For mRNA, poly(A) can increase the translation efficiency through cooperation with the 5′ cap mediated by some host factors [15]. For potyviruses, poly(A) can synergisticly enhance the translation mediated by 5’UTR of TEV [22] However, the influence of poly(A) on the translation of WYMV RNA is not clear and the effect of poly(A) on the minus strand synthesis from WYMV RNA also remains to be investigated.

In this study, variable polyadenylation including the absence of poly(A) tail at the 3′ end of WYMV RNAs was found from wheat naturally infected by WYMV in China. In vitro assays showed that variable polyadenylation differentially regulated the translation and minus strand synthesis of WYMV. It is implied that the variable polyadenylation may regulate the template selection for translation and replication or the molecular transition between translation and replication of WYMV.

## Materials and methods

### 3’RACE

Total RNA was extracted from wheat leaves showing yellow mosaic and dwarf symptoms at Linyi, Shandong province, China in 2015. Firstly, the adaptor (5-aaaaaaaaaaaaaaaaagcttgagctcgagtcctcgtcactctgctcactgg-3) was linked with the 3′ end of total RNA using T4 RNA ligase (New England Biolabs). Reverse transcription on the adapted total RNAwas performed using M-MLV reverse transcriptase (Takara) and primer Q_0_ (5-ccagtgagcagagtgacg-3), which complements with the terminal sequence of the adaptor. RT products were then used as templates to clone the 3′ terminal sequences using LA-Taq polymerase (Takara) with primers Q_0_ and WYM-R1–7264-F (5-gctgctttaggcacaggtac-3). RT-PCR products were cloned into pMD18-T (Takara) and sequenced using the primer WYM-R1–7264-F.

### Plasmids and DNA fragments

All plasmids were constructed based on the firefly luciferase reporter construct pT7-F-3’UTRssp vector [28] and confirmed by DNA sequencing. pFluc-WY-R1-5 U was constructed by inserting the 5’UTR (162 nt) of WYMV RNA1 (GenBank: AF067124) between the T7 promoter and firefly luciferase (*Fluc*) gene in the pT7-F-3’UTRssp vector using BamH I and Sma I; the in vitro transcript from this plasmid was designated F-WY-R1-5 U. pFluc-WY-R1-3 U and pFluc-WY-R1-3 U·15A, with or without 15 adenylates, respectively, were separately constructed by inserting the 3’UTR (258 nt) of WYMV RNA1 (GenBank: AF067124) downstream of the *Fluc* gene in the pT7-F-3’UTRssp vector using Nru I and Ssp I; the in vitro transcript from these plasmids were designated F-WY-R1-3 U and F-WY-R1-3 U·15A. pFluc-WY-R1-5 U-3 U and pFluc-WY-R1-5 U-3 U·15A, without and with 15 adenylates, respectively, were separately constructed by inserting 3’UTR of WYMV RNA1 (GenBank: AF067124) downstream of the *Fluc* gene in pFluc-WY-R1-5 U. The 5’UTR (171 nt) and 3’UTR(768 nt) of WYMV RNA2 (GenBank: AF041041) with or without 15 adenylates were inserted at the same sites as that of RNA1 in pT7-F-3’UTRssp vector to make pFluc-WY-R2-5 U, pFluc-WY-R2–3 U, pFluc-WY-R2–3 U·15A, pFluc-WY-R2-5 U-3 U and pFluc-WY-R2-5 U-3 U·15A.

DNA fragments with upstream T7 promoter were amplified using *pfu Taq* polymerase (Takara) and corresponding primers (Additional file 1: Table S1). DNA fragments include RNA1-3 U·0A, RNA1-3 U·15A, RNA1-3 U·30A, RNA2–3′·0A, RNA2–3′·15A, RNA2–3′·30A.

### In vitro transcription with T7 RNA polymerase

In vitro transcription was performed using T7 RNA polymerase to make RNA based on the template of linearized plasmids or DNA fragments. The 100 μL reaction consisted of DNA template 12 pmol, 10 mM DTT, 0.5 mM ATP, 0.5 mM GTP, 0.5 mM CTP, 0.5 mM UTP, T7 RNA polymerase 25 U, 2X T7 buffer 50 μL and ddH_2_O. In vitro transcription was performed at 37 °C for 2 h.

RNAs made from linearized Fluc reporter plasmids were used in subsequent in vitro translation assays. RNAs synthesized from DNA fragments were used as template to perform in vitro replication mediated by the WYMV NIb protein.

### In vitro translation assays

In vitro translation assays were performed as previously described [28]. Briefly, 3 pmol of in vitro-synthesized RNA transcripts from designated translation reporter constructs were used for a 25 μl translation reaction using wheat germ extracts (WGE; Promega) according to the manufacturer’s instructions. The luciferase activity was measured by using a luciferase assay reporter system (Promega) and a Modulus microplate multimode reader (Turner BioSystems). At least three independent in vitro translation assays were performed for each construct.

### Expression and purification of WYMV NIb protein

NIb coding sequences of WYMV HC isolate (GenBank. AF067124) was amplified by primers WYM-R1–4921-BamHI-F (5-atggatccatggcctccgacactctcagc-3) and WYM-R1–6504-SalI-R (5-atgtcgacgatagtttggagctcaatgctgct-3). PCR products were digested by BamH I and Sal I and inserted into pMAL-C2X (NEB) with T4 DNA ligase (Takara). The positive plasmid of pMAL-WY-NIb was transformed into *E. coli* Rossetta. The WYMV NIb proteins can be expressed under the induction of 0.5 mM IPTG at 37 °C. The WYMV NIb proteins were purified using amylose resin (NEB E8021S) by affinity column chromatography according to the manufacturer’s instructions.

### In vitro replication mediated by WYMV NIb protein

The 50 μL reaction system consisted of 8 pmol of RNA templates, 0.1 M Tris-Cl (pH 8.2), 0.01 M MgCl_2_, 0.01 M DTT, 0.11 M KCl, 17.5 μg yeast tRNA, 1 mM ATP, 1 mM GTP, 1 mM CTP, 0.01 mM UTP, 5 UCI α-32P-UTP (PerlinElmer), 12.5 μg of purified WYMV NIb protein and ddH_2_O. The in vitro replication reaction was performed at 20 °C for 1.5 h. After the reaction, 70 μL ddH_2_O and 120 μL of phenol/chloroform were added, followed by centrifugation at 13,000 rpm. The supernatant was precipitated by adding 2.4 volumes of NH_4_Ac / isopropanol (5 M NH_4_Ac: isopropanol = 1: 5) and placed at − 20 °C for at least 2 h. The precipitate was resuspended using 10 μL RNA loading buffer and separated in 5% PAGE gel containing 8 M urea with 1500 mA for 1.5 h. The gel was dried and exposed to a phosphorimager screen, followed by detection with the Typhoon FLA-7000 (GE Healthcare).

## Results

### Variable adenylation at the 3′ end of WYMV RNAs

**Fig. 1:**
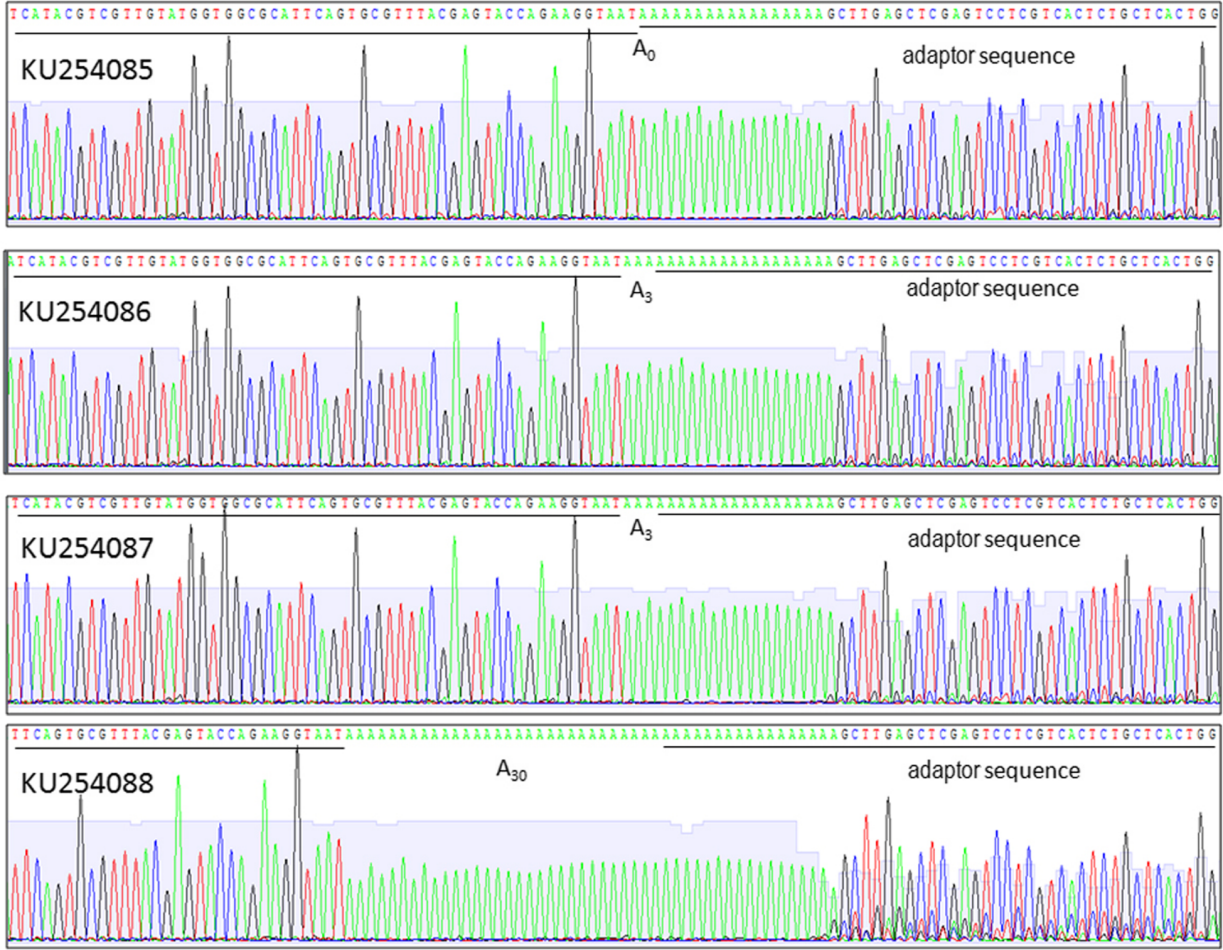
3’RACE for WYMV RNA 1. Adaptor sequences and 3′ terminal sequences of WYMV RNA1 are underlined and potential poly(A) is located between them. A_0_ indicates absence of poly(A), A_3_ indicates three adenylates at the 3′ end, A_30_ indictes thirty adenylates at the 3’end

**Fig. 2:**
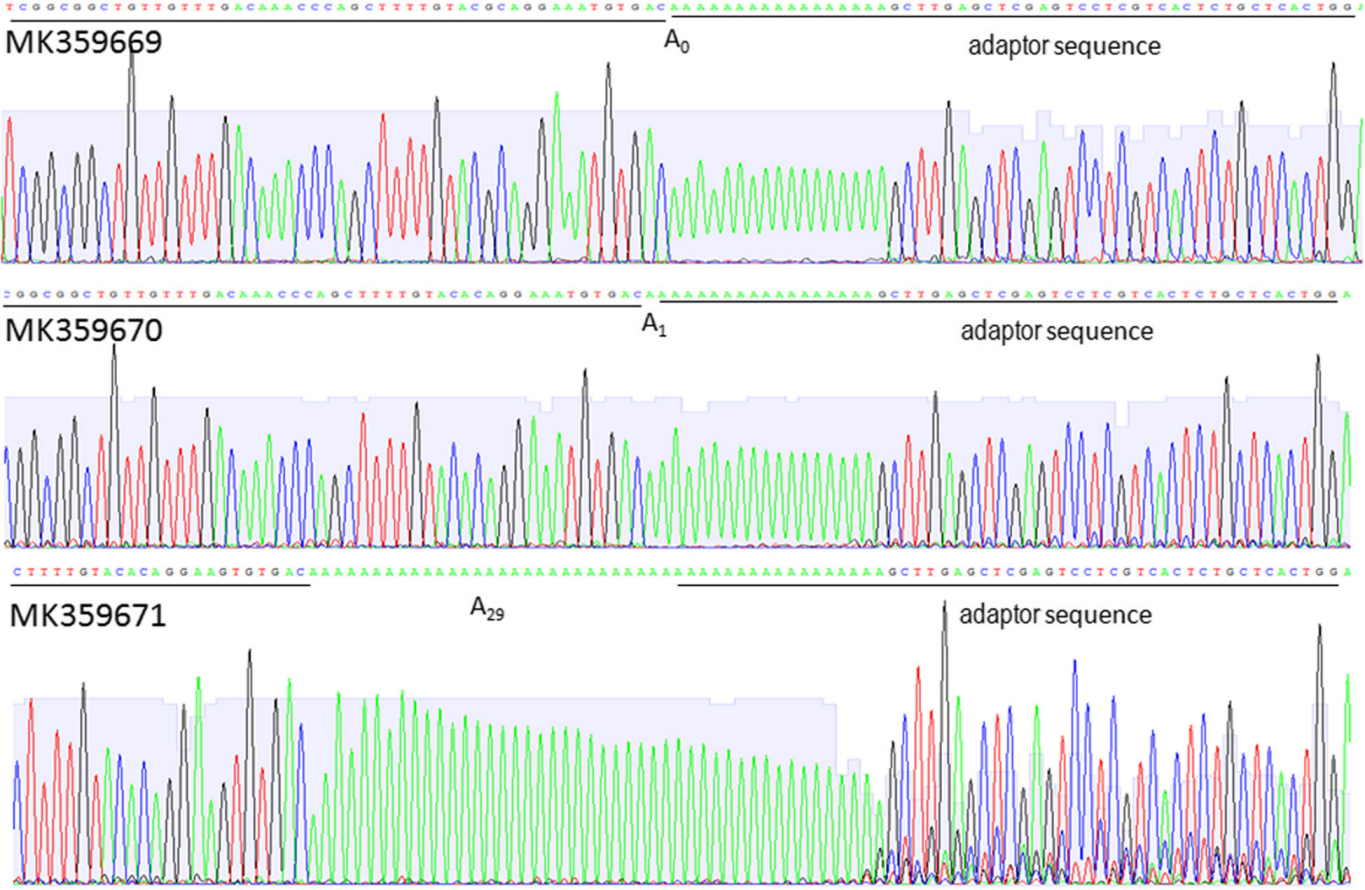
3’RACE for WYMV RNA2. Adaptor sequences and 3′ terminal sequences of WYMV RNA2 are underlined and poly (A) is located between them. A_0_ indicates absent of poly (A), A_1_ indicates one adenylates at the 3′ end, A_29_ indicates twenty-nine adenylates at the 3’end

In this study, status of the 3′ polyadenylation in WYMV RNA1 was analyzed for the first time. Total RNAs were extracted from wheat showing yellow mosaic and dwarf symptoms in fields at Shandong province, China in 2015. RT-PCR was performed to confirm the existence of WYMV (data not shown). An aliquot of the same total RNAs was used in 3’RACE to clone the 3′ terminal and potential poly(A) sequences. Sequencing data of four clones showed the differential polyadenylation at the 3′ end of WYMV RNA1. One clone (GenBank: KU254085) lacked adenylation at the 3′ end, two clones (GenBank: KU254086, KU254087) had three adenylates at the 3′ end, and one clone (GenBank: KU254088) had 30 adenylates at the 3′ end (Fig. 1). Although the lengths of poly(A) at the 3′ ends of these four clones are different, 3′ UTR sequences excluding poly(A) showed a high identity at 99.22% among the clones(Table 1). The clones also shared high identities (97.67 - 98.45%) with previously reported Chinese isolates of WYMV RNA1 (GenBank: FJ361764, FJ361765 and FJ361766) (Table 1). 3’RACE was also used to analyze the 3’terminal sequences of WYMV RNA2. One clone (MK359669) lacked adenylation at the 3’end, one clone (MK359670) had one adenylates at the 3′ end, and one clone (MK359671) had 29 adenylates at the 3′ end (Fig. 2). To the best of our knowledge, this is the first report of WYMV RNA without 3′ poly(A).

**Table 1:** Nucleotide identity (%) among 3′ UTR of different WYMV RNA1

	KU254085	KU254086	KU254087	KU254088	FJ361764	FJ361765	FJ361766
KU254085	100	99.22	99.22	99.22	98.07	97.67	98.45
KU254086	99.22	100	99.22	99.22	98.07	97.67	98.45
KU254087	99.22	99.22	100	99.22	98.07	97.67	98.45
KU254088	99.22	99.22	99.22	100	98.07	97.67	98.45

Note: Identity analysis was performed with the potential poly(A) sequences omitted

In order to estimate the ratio of clones with different lengths of poly(A), an additional 96 clones were randomly selected and the length of 3′ poly(A) of WYMV RNA1 was analyzed. The 3′ poly(A) in these 100 clones ranged from 0 to 33 adenylates in length (Table 2). Clones A_0_ lacking poly(A) accounted for 27, 46% of clones A_1_-A_5_ had 1 to 5 adenylates, and 20% of clones A_6_-A_10_ had 6 to 10 adenylates (Table 2). The remaining 7% was the clones with more than 25 adenylates (Table 2). The results suggest that WYMV RNA has different lengths of 3′ poly(A) in vivo which presents the pattern of polarization .

**Table 2:** Statistics of different lengths of poly(A) at the 3’end of WYMV RNA1s in this study

	A_0_	A_1_-A_5_	A_6_-A_10_	A_11_-A_20_	A_21_-A_25_	A_26_-A_30_	A_33_	total
Amount	27	46	20	0	0	6	1	100
Ratio	27%	46%	20%	0%	0%	6%	1%	100%

Note: Subscripted numbers indicate the number of adenylates at the 3′ end of WYMV RNA1

### 3′ polyadenylation had oppositive effects on in vitro translation mediated by UTRs

Polyadenylation plays an essential role the translation of mRNA through the cyclization of mRNA, a process which also involves the 5′ cap [15]. However, WYMV lacks the 5′ cap and has a VPg protein at the 5′ end of its RNA.

**Fig. 3:**
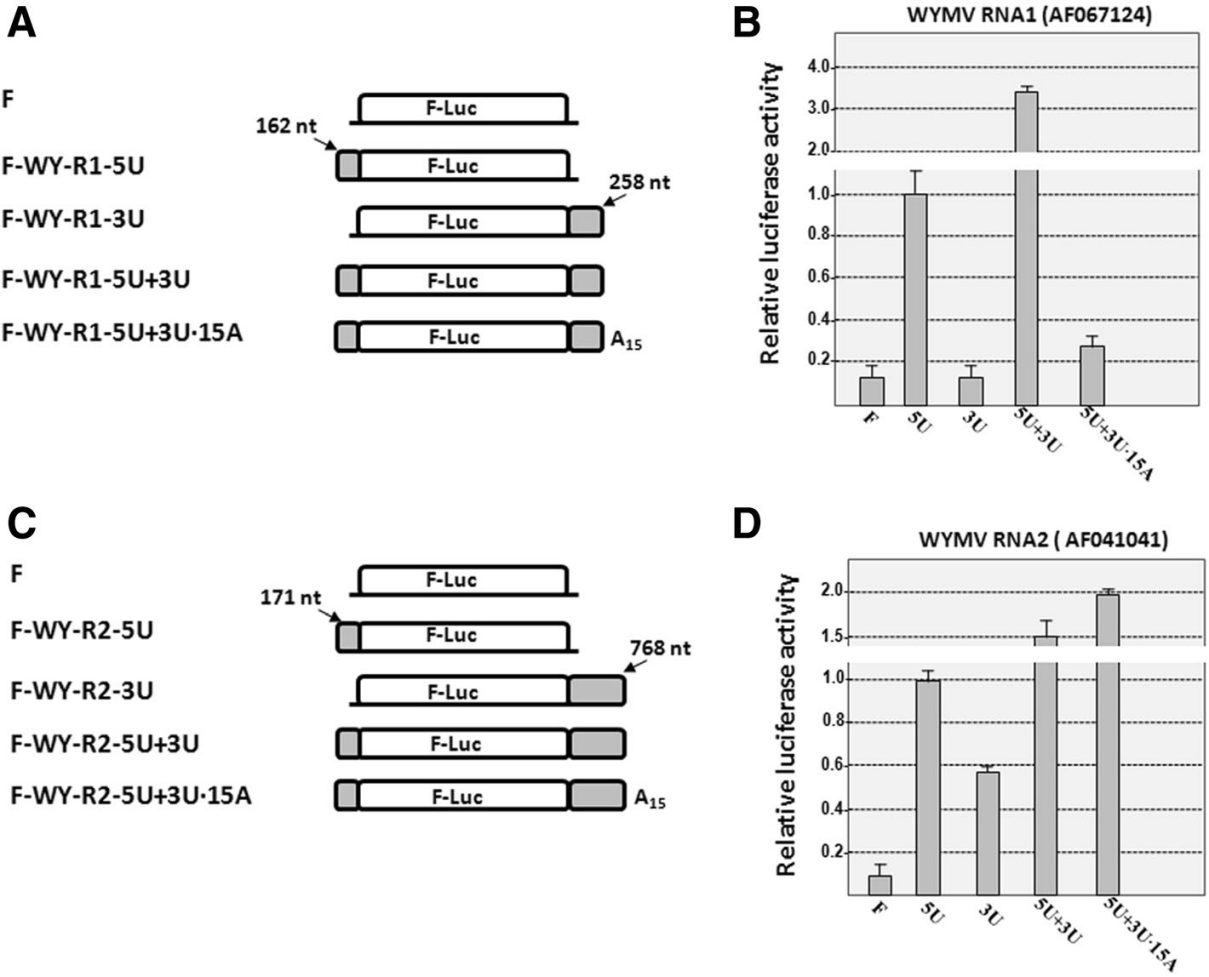
Effect of polyadenylation on in vitro translation mediated by WYMV UTRs. **a** Constructs of firefly luciferase reporter vector containing UTRs of WYMV RNA1. **b** Effect of UTRs of WYMV RNA1 with or without poly(A) on translation of F-luc. **c** Constructs of firefly luciferase reporter vector containing UTRs of WYMV RNA2. **d** Effect of UTRs of WYMV RNA2 with or without poly(A) on translation of F-luc. 5’UTR and 3’UTR of WYMV RNA1 (AF067124) is 162 nt and 258 nt, respectively; 5’UTR and 3’UTR of WYMV RNA2 (AF041041) is 171 nt and 768 nt, respectively. F-luc indicates firefly luciferase coding region; shadow boxes indicate corresponding WYMV UTRs

For WYMV, the effect of 3′ polyadenylation on translation was evaluated using RNA lacking the 5′ cap and VPg (Fig. 3). Firstly, the effect of UTRs of WYMV RNA1 or RNA2 on translation of *Fluc* reporter gene was tested. Compared with the mock vector, the presence of only the 5′ UTR of WYMV RNA1 or RNA2 enhanced translation of Fluc to 8 fold or 10 fold, respectively (Fig. 3B and D). The presence of only the 3’UTR of WYMV RNA1 had no effect on translation (Fig. 3B), while the 3’UTR of WYMV RNA2 alone enhanced translation of Fluc to 5.6 fold (Fig. 3D). The positive effects of 5′ UTR of WYMV RNA1 and RNA2 on Fluc translation can be further enhanced to 3.3 fold and 1.5 fold, respectively, by addition of the corresponding 3’UTR (Fig. 3B and D). It is suggested that 5′ UTR of WYMV RNA1 or RNA2 can enhance the activity of cap-independent translation and 3′ UTR further promotes the positive activity on translation, possibly through potential long-distance RNA-RNA interaction.

Subsequently, the effect of poly(A) on translation of corresponding constructs was tested. An additional 15 adenylates on F-WY-R1-5 U + 3 U decreased the translation of Fluc to 9.1% of F-WY-R1-5 U + 3 U (Fig. 3B). Addition of 15 adenylates to F-WY-R2-5 U + 3 U increased the translation of Fluc to 1.3 fold of F-WY-R2-5 U + 3 U (Fig. 3D). These opposite effects of 3′ polyadenylation may be associated with the different effects of 3’UTR of RNA1 and RNA2 on translation (Fig. 3B and D). These results suggest that polyadenylation of the WYMV RNA1 and RNA2 has opposite effects on their own cap-independent translation mediated by corresponding UTRs.

### 3′ polyadenylation had negative effect on in vitro replication mediated by WYMV NIb

**Fig. 4:**
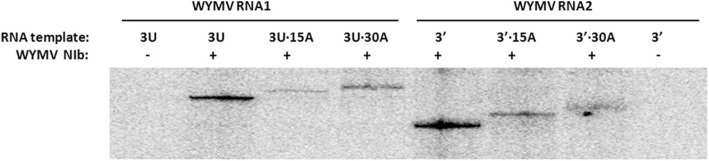
Effect of polyadenylation on in vitro replication mediated by WYMV NIb. 3 U indicates 3′ UTR of WYMV RNA1 (GenBank: AF067124) , 3 U•15A and 3 U•30A indicate 3′ UTR of WYMV RNA1 (GenBank: AF067124) with 15 adenylates or 30 adenylates, 3′ indicates 3′ terminal 192 nt fragment of WYMV RNA2 (GenBank: AF041041), 3′•15A and 3′•30A indicate 3′ terminal 192 nt fragment of WYMV RNA2 (GenBank: AF041041) with 15 adenylates or 30 adenylates

In addition to its role as the template for translation, viral RNA of WYMV is also the template for synthesis of the complementary strand, which then is transcribed into the progeny viral RNAs. The effects of alternative polyadenylation on replication were tested using an in vitro replication system mediated by WYMV NIb, which has RNA-dependent RNA polymerase (RdRp) activity [29]. WYMV NIb can synthesize the complementary strand from RNA1 3′ UTR (3 U) or RNA2 3′ terminal 192 nt (3′) (Fig. 4). When 15 or 30 adenylates were added to the RNA1 3′ UTR (3 U) or RNA2 3′ terminal 192 nt (3′), the production of complementary transcripts from the corresponding template declined greatly (Fig. 4). It is suggested that additional adenylates may greatly inhibit the synthetic efficiency of complementary strand from RNA1 or RNA2 of WYMV (Fig. 4). In addition, length of complementary strands was equal to that of the corresponding templates (Fig. 4). This indicates that polyadenylation of RNA1 or RNA2 3′ UTR is stable during in vitro replication.

## Discussion

As small infectious entities replicating only in the living cell, viruses must evolve some properties so that they can exploit the translation machinery of the host to survive. mRNAs in eukaryotes are co-transcribed to obtain a non-templated poly(A) tail of about 250–300 adenosine residues, which contributed to the regulation of the stability, transport and translation of mature transcripts [13, 30]. Many RNAs of RNA viruses have poly(A) tails, whose length is shorter than that of mRNAs [11]. The polyadenylated tail of viral RNA has been considered a factor in RNA stability and translation and mimics the role of the stable poly(A) tail in eukaryotic mRNAs [31, 32]. In addition, variable polyadenylation at the 3′ end of of some viruses’ RNA, including WYMV in this study, has been detected [16,]. These variable polyadenylation tails in RNA viruses may be the direct transcriptional products from different poly(U) segments in the template strands [6, 8, 33, 34] or may undergo the degradation of the poly(A) tail by a 3′ exonuclease like mRNA decay [35, 36].

Alternative polyadenylation in RNA viruses should have more complex roles than in mRNAs since RNA from RNA viruses can serve as the template for both translation and replication [19]. In this study, the effects of alternative polyadenylation on translation and replication of WYMV were investigated. At the translation level, the cap-independent activity or IRES activity of 5′ UTR of WYMV RNA1 and RNA2 was identified using Fluc-fused constructs. The cap-independent activity of 5′ UTR of WYMV RNAs can be further enhanced by corresponding 3′ UTR without adenylates through potential RNA-RNA interaction (Fig. 3). This provides new evidence about the IRES activity of 5′ UTR in VPg-containing RNA viruses. For picornaviruses, VPg serves as a primer to synthesize the nascent viral RNA and exists at the 5′ end [37]. This VPg can be released from viral RNA though “unlinkase” activity via TDP2 enzyme [38]. For RNA viruses encoding VPg, viral RNAs with or without VPg can achieve effective translation [39, 40]. It is suggested that the presence of VPg at the 5′ end of viral RNA is not essential for effective translation.

Additional poly(A) showed opposite effects on translation mediated by WYMV RNA1 or RNA2 UTRs (Fig. 3). However, the poly(A) tail of mRNA acts synergistically with the 5′ cap to facilitate translation initiation through stabilization of the closed loop formed by the cap structure bound to the translation initiation factor 4F (eIF4F) complex [1, 41]. It is suggested that 3’poly adenylation can have a negative effect on cap-independent translation. In addition, it is also implied that the cap-independent translation mediated by WYMV RNA1 or RNA2 UTRs should have different features, such as the requirement for translation initiation factors. This alternative polyadenylation of WYMV RNA may provide multiform selection on the template for the translation of WYMV RNA1 or RNA2.

In addition to being the template for translation, RNAs of RNA viruses also serve as the templates of their complementary strands during the replication of viral genomes. Previous studies on genome replication of RNA viruses were focused on characteristics of the core promoters and other cis-elements [42–44]. The function of polyadenylation during the synthesis of complementary strands of viral RNA was usually ignored, although many RNA viruses possess the 3′ polyadenylation. In this study of WYMV, 3′ terminal fragments of RNA1 or RNA2 served as good templates for the effective synthesis of their complementary strands in in vitro replication system mediated by NIb protein of WYMV, (Fig. 4). However, addition of 15 or 30 adenylates remarkably inhibited the synthesis of the complementary strands from the corresponding templates. It is suggested that 3′ polyadenylation had a negative effect on the synthesis of minus strands of WYMV RNAs. In this study, effect of polyadenylation on translation and transcription of WYMV was analyzed in vitro. It is suggested that WYMV RNAs containing different length of poly(A) may be responsible for translation or replication of WYMV in vivo and ensure suitable infectivity of WYMV. However, effect of different polyadenylation on WYMV infectivity can not be tested due to shortage of WYMV infectious clone.

The detailed mechanism of alternative polyadenylation of RNA viruses remains unknown. If alternative polyadenylation tails are the direct transcriptional products from different poly(U) segments in the template strands, this alternative polyadenylation on WYMV RNAs could provide suitable templates for the different requirements of translation and replication. If alternative polyadenylation is caused by RNA decay, as is the case with mRNA, the change of polyadenylation of viral RNAs may be responsible for the transition between translation and replication. The transition from translation to replication for RNA viruses without polyadenylation may be regulated by the structural change of cis-elements at the 3′ end of viral RNA due to the binding of RdRp [20]. In summary, findings of this study on WYMV have provided insights into understanding new functions of 3′ polyadenylation in addition to those on mRNAs.

## Conclusions

3′ end of WYMV RNAs present variable polyadenylation even no polyadenylation. 3′ polyadenylation have opposite effect on translation mediated by UTRs of WYMV RNA1 or RNA2. 3′ polyadenylation have negative effect on minus-strand synthesis of WYMV RNA in vitro. Variable polyadenylation of WYMV RNAs may provide sufficient selection on the template for translation and replication.

## Additional file

Additional file 1: **Table S1.** The primers used in this study. (DOCX 15 kb)

## Abbreviations

Fluc: firefly luciferase
kb: kilobase
kD: kilo dalton
RACE: rapid-amplification of cDNA ends
RdRp: RNA-dependent RNA polymerase
RT-PCR: Reverse Transcription - Polymerase Chain Reaction
UTR: untranslated region
WGE: wheat germ extract
WYMV: *Wheat yellow mosaic virus*
