# Supplementary material for: Variable 3’polyadenylation of Wheat yellow mosaic virus and its novel effects on translation and replication
Source: Virol J. 2019 Feb 20;16:23. doi: 10.1186/s12985-019-1130-z (PMC6383263; doi:10.1186/s12985-019-1130-z)
Supplement: Supplementary file 1 — Table S1. The primers used in this study. (DOCX 15 kb) [file 12985_2019_1130_MOESM1_ESM.docx]

**Additional file 1: Table S1** The primers used in this study

| Primer name | Sequence (5' to 3') | purpose |
| --- | --- | --- |
| WYM-R1-7264-F | GCTGCTTTAGGCACAGGTAC | 3’RACE |
| WYM-R2-3313-F | TCTTTCAAATATCTCGGCGGC | 3’RACE |
| 3-race-1 | (A)_17_GCTTGAGCTCGAGTCCTCGTCACTCTGCTCACTGG | 3’RACE |
| Qo | ccagtgagcagagtgacg | 3’RACE |
| WYM-R-1-BamH I-F | ATGGATCCAAAATAAAACCACCACAAAC | pFluc-WY-R1(2)-5U-3U(3U·15A) |
| WYM-R1-Sma1-162-R | ATCCCGGGCTCGAAGGGAGTGAAGAA | pFluc-WY-R1-5U-3U(3U·15A) |
| WYM-R1-7387-Ssp1-F | ATAATATTACCATAACCCCCCTCC | pFluc-WY-R1-3U(3U·15A) |
| WYM-R1-7644-Ssp1-R | ATAATATTATTACCTTCTGGTACTCGTA | pFluc-WY-R1-3U |
| WYM-R1-7644A-Ssp1-R | ATAATATT(T)_15_ATTACCTTCTGGTACTCGTAAACGCAC | pFluc-WY-R1-3U·15A |
| WYM-R2-170-Sma1-R | ATCCCGGGAGAAGCGGAAAGAACTAGG | pFluc-WY-R2-5U-3U(3U·15A) |
| WYM-R2-2884-Ssp1-F | ATAATATTTTTTCCGCATTTGCTCATTC | pFluc-WY-R2-3U(3U·15A) |
| WYM-R2-3652-Ssp1-R | ATAATATTGTCACATTTCCTGTGTACA | pFluc-WY-R2-3U |
| WYM-R2-3652A-Ssp1-R | ATAATATT(T)_15_GTCACATTTCCTGTGTACAAAAGCTGG | pFluc-WY-R2-3U·15A |
| WYM-T7-R1-7387-F | AT*taatacgactcactata*GGACCATAACCCCCCTCCGC | RNA1-3U·0A(15/30A) |
| WYM-R1-7644-R | ATTACCTTCTGGTACTCGTAAACGCAC | RNA1-3U·0A |
| WYM-R1-7644-15A-R | (T)_15_ATTACCTTCTGGTACTCGTAAACGCAC | RNA1-3U·15A |
| WYM-R1-7644-30A-R | (T)_30_ATTACCTTCTGGTACTCGTAAACGCAC | RNA1-3U·30A |
| WYM-T7-R2-3460-F | AT*taatacgactcactata*GGGCACCGCGCGTTGTGCCACG | RNA2-3’·0A(15/30A) |
| WYM-R2-3652-R | GTCACATTTCCTGTGTACAAAAGCTGG | RNA2-3’·0A |
| WYM-R2-3652-15A-R | (T)_15_GTCACATTTCCTGTGTACAAAAGCTGG | RNA2-3’·15A |
| WYM-R2-3652-30A-R | (T)_30_GTCACATTTCCTGTGTACAAAAGCTGG | RNA2-3’·30A |

Note: Italic nucleotides indicating T7 promoter; Underlined nucleotides indicating enzymes.
